# Patient Diagnosed Initially with Peripartum Cardiomyopathy, Later Rediagnosed with Peripartum Myocardial Infarction: A Case Report

**DOI:** 10.3390/life15101502

**Published:** 2025-09-24

**Authors:** Spas Kitov, Maria-Florance Kitova, Meri Hristamyan, Lyudmila Vladimirova-Kitova

**Affiliations:** 1 I-st Department of Internal Diseases, Section of Cardiology, Faculty of Medicine, Medical University of Plovdiv, 4001 Plovdiv, Bulgaria; spas.kitov@mu-plovdiv.bg; 2 Clinic of Cardiology, St. George University Hospital, 4002 Plovdiv, Bulgaria; 3 Faculty of Medicine, Medical University of Plovdiv, 4001 Plovdiv, Bulgaria; mimikitova7@abv.bg; 4 Department of Epidemiology and Disaster Medicine, Faculty of Public Health, Medical University of Plovdiv, 4001 Plovdiv, Bulgaria; meri.hristamyan@mu-plovdiv.bg

**Keywords:** peripartum cardiomyopathy, pregnancy-associated myocardial infarction, cardiac magnetic resonance, multimodality imaging

## Abstract

Differentiating peripartum cardiomyopathy (PPCM) from pregnancy-associated myocardial infarction (PAMI) is challenging due to shared risk factors. We report a case of a 35-year-old woman who suffered a seizure and cardiac arrest in the final month of her second pregnancy. Echocardiography showed a normal left ventricular ejection fraction (LVEF). Three days later, she developed heart failure symptoms and a marked reduction in LVEF. After one month of treatment, LVEF nearly normalized, but regional wall motion abnormalities subsequently appeared, prompting coronary angiography, which showed normal coronary arteries. Thus, PPCM was diagnosed. One year later, cardiac magnetic resonance imaging, performed due to her wish for another pregnancy, showed a scar consistent with a previous transmural myocardial infarction. We interpret this as a case of PAMI. Despite medical contraindications, she became pregnant one year after the infarction and delivered via C-section. Five years post-event, there are no signs of heart failure. This case lies in the gray zone of pregnancy-related cardiac complications and highlights the importance of multimodality imaging for thorough structural and functional assessment.

## 1. Introduction

Peripartum cardiomyopathy (PPCM), sometimes also called pregnancy-associated or postpartum cardiomyopathy, is a rare idiopathic heart condition characterized by overt heart failure resulting from impaired contraction of the left ventricle, with an ejection fraction less than 45%, which may occur with or without enlargement of the left ventricle [1]. Not much is certain about the mechanisms leading to PPCM besides its strong time correlation with pregnancy [2]. It designates a pathology that happens in the last months of pregnancy, up to five months post-delivery [3,4,5].

PPCM affects approximately 1 in 1000 to 1 in 5000 live births worldwide, with higher incidence reported in African and African-American women (1 in 102 cases in Nigeria) [6]. Risk factors include advanced maternal age (35+), hypertensive disorders of pregnancy, multiple gestations, and race [2,7].

Pregnancy-Associated Myocardial Infarction (PAMI) is a heart attack that occurs during pregnancy or the postpartum period, and it is a significant cause of maternal cardiac morbidity and mortality [8], with some authors reporting mortality related rates being around 4.5% [9]. The mechanism for development in a large number of these cases is different from other cases of ischemic heart disease and is most likely linked to coronary artery spasm associated with a probable hypercoagulability state (identified thrombophilic mutations), and undergoing spontaneous fibrinolysis [10,11]. There is also the possibility that the mechanism of myocardial infarction may be based on high levels of progesterone.

PAMI occurs in approximately 2.8 to 8.1 cases per 100,000 deliveries, which is about four times higher than in non-pregnant women of reproductive age, with the incidence increasing, likely due to better detection and more women with cardiovascular risk factors becoming pregnant [8]. Significant risk factors include advanced maternal age (35+), black race, infections, hypertensive disorders of pregnancy, and traditional cardiovascular risk factors such as smoking and hyperlipidemia [9].

PPCM and PAMI share common risk factors, with the risk always higher in following pregnancies, and do not have a completely studied pathogenesis. Despite many scientific breakthroughs in the last two years, this area is still not yet fully elucidated [12,13].

PPMC can result in severe complications including heart failure decompensation, thromboembolic events, arrhythmias, need for mechanical circulatory support, as well as potential cardiac transplantation, Delays in the diagnosis and severely reduced left ventricular ejection fraction (<25%) significantly increase risks of major adverse events and mortality [14]. PAMI also carries risks of maternal mortality, heart failure, arrhythmias, and increased rates of adverse pregnancy outcomes such as preterm birth and stillbirth [15].

Considering all these factors, the decision of a new pregnancy should be an individual one. In clinical practice, this is a very difficult decision for the cardiologist, in the absence of contraindications from the obstetrician-gynecologists. In this case report, we present a case of a young woman with an unremarkable past medical history, who suffered a seizure and a cardiac arrest in the final month of her second pregnancy, leading to initial diagnosis of PPCM, later changed to PAMI.

## 2. Case Report

### 2.1. Anamnesis

The following case report concerns a 38-year-old woman (as of 2018), who had an uncomplicated pregnancy at the age of 24. She is an active smoker but denies smoking while pregnant, denies use of oral contraceptives, and reports normal blood pressure. Family history shows no evidence of premature cardiovascular disease, sudden cardiac death, or preeclampsia; the patient reports relatives with arterial hypertension in advanced age. In 2014, at the age of 35, she became pregnant for a second time. The pregnancy had an unremarkable course until the beginning of the ninth lunar month when she developed a respiratory tract infection. About a week later (December 2014), she sought medical attention at a local outpatient clinic because of pain in her chest and back that had lasted for about 40 min.

### 2.2. Physical Examination

Physical examination at the time of presentation revealed blood pressure (BP) 110/75 mmHg, rhythmic heart rate 74/min, normal heart sounds, normal vesicular breathing, and no signs of peripheral edema. During an attempt to perform electrocardiography (ECG), the patient suffered a generalized seizure, lost consciousness, did not resume spontaneous breathing, and eventually developed ventricular fibrillation. She was successfully resuscitated after cardiopulmonary resuscitation, including three 360 J defibrillations, and was transported intubated and on catecholamine infusion to the Department of Reanimation and Intensive Care at University General Hospital for Active Treatment “St. George” Plovdiv. Her condition on admission was critical, characterized by unstable hemodynamics (BP 90/50 mmHg on dopamine infusion; sinus tachycardia approximately 160/min), and bilateral pulmonary congestion. She experienced several episodes of sustained ventricular tachycardia, idioventricular rhythm, and ventricular fibrillation. Chest X-ray showed high-grade pulmonary edema.

### 2.3. Laboratory Examination

Laboratory tests demonstrated the critical state of the patient in the emergency room: hemoglobin—114 g/L, white blood cells—47.6 × 10^9^/L, hematocrit—0.33, lactate dehydrogenase—970 U/L, alanine transaminase—186 U/L, aspartate aminotransferase—158 U/L, creatine kinase—631 U/L, creatine kinase-MB—172 U/L, troponin I—25.46 ng/L, potassium—4.7 mmol/L, chloride—101 mmol/L, magnesium—0.28 mmol/L, calcium—1.92 mmol/L, urine—protein 3 (+).

Brain computer tomography was performed and revealed an old calcification in the left occipital lobe, which was interpreted by both the radiologist and the consulting neurologist as having no evident connection to the current event

### 2.4. Echocardiography

**Figure 1:**
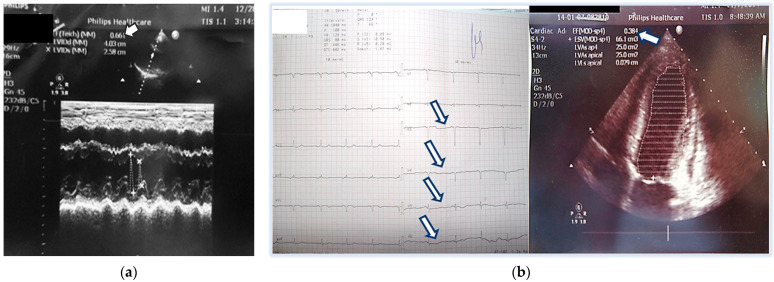
(**a**) Initial echocardiography at the emergency room—normal ejection fraction = 66%; (**b**) ECG (pathological progression of the R wave in the precordial series) and Echocardiography three days later at the Intensive Care Unit—with significantly reduced ejection fraction up to 38%. Arrows show pathological progression of the R wave in the precordial series.

The patient was consulted by a cardiologist, and echocardiography was performed, which was within normal limits: Left atrium—32 mm, ascending aorta—33 mm, right ventricular diameter—27 mm; septum and posterior wall—9 mm; telediastolic diameter (TDD)—40 mm, telesystolic diameter (TSD)—25 mm, left ventricle ejection fraction (LVEF) (Teicholz)—66%; anterior mitral leaflet prolapse with mitral regurgitation 0+; no pericardial effusion. Systolic pressure of the pulmonary artery—normal (Figure 1a).

Simultaneously, she was consulted by an obstetrics-gynaecology specialist, and the exam showed intrauterine death of the fetus, which necessitated emergency Caesarean section, with removal of a 2900 g fetus. The intrauterine death was attributed to the hemodynamic instability of the mother.

The patient’s condition improved, and the next day she was extubated and hemodynamically stable. The official diagnosis was eclampsia complicated by pulmonary edema, although it was stated that the electrolyte disorder—hyperkalemia and extremely low magnesium levels—could not be ruled out as the cause of the seizure and rhythm disturbances. Follow-up echocardiography was performed but showed similar findings and no new abnormalities.

The ECG recorded during her stay at the Intensive Care Unit was abnormal, showing left posterior fascicular block and pathological R-wave progression in the precordial leads (Figure 1b). The latter was associated with a pseudoinfarction pattern due to the dilated left ventricle in PPCM. An ECG recording of the same patient done 2 years prior showed normal QRS axis and normal R-wave progression in the precordial leads.

Three days later, she reported dyspnea and fatigue on minimal physical effort, accompanied again by back pain. Echocardiography showed reduced LVEF (38% by Simpson method) with global hypokinesis, the appearance of tricuspid regurgitation, and elevated pulmonary pressure. A slight dilation of the left chambers compared to previous findings was noted: TDD 5.48 cm, TSD 4.03 cm; TDV 108 mL; TSV 66 mL; LVEF 38% (Simpson) (Figure 1b).

At that time, these findings were deemed consistent with PPCM [16,17]. Immediate treatment with torasemide 5 mg, carvedilol 2 × 3.125 mg, and ramipril 2.5 mg was started. Ejection fraction improved to approximately 44% within one week, with no reduction in left ventricular dimensions. The patient’s functional class also improved, and she was eventually discharged.

**Figure 2:**
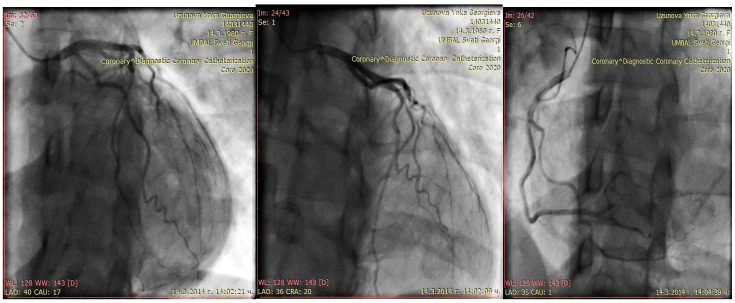
Coronary angiography performed 1 months after the initial event—normal coronary anatomy: no cardiac sighs, EchoC-normal EF, but persist regional apical wall motion abnormality; diagnosis—periportal cardiomyopathy; treatment with torasemide 5 mg, carvedilol 2 × 3.125 mg, and ramipril 2.5 mg.

A month later, she became symptomatic again. Echocardiography revealed the appearance of segmental wall motion abnormalities—a hypo- to dyskinetic zone in the latero-apical segments, while left ventricular dimensions and EF remained similar (TDV 127 mL; TSV 62 mL; EF 44% Simpson; TDD 57 mm; TSD 44 mm; EF 45% Teichholz; RV 25 mm; anterior mitral valve prolapse with mitral regurgitation grade I; tricuspid regurgitation grade I+; normal systolic pressure in the pulmonary artery, 30 mmHg). Since segment abnormalities are not a typical finding in PPCM and because her initial presentation was predominantly precordial and back pain coronary angiography was recommended and performed 1 months after the initial event. It showed normal coronary anatomy, thus making the diagnosis PPCM likely. (Figure 2) In the differential diagnostic plan, spontaneous dissection of the coronary arteries was considered. During the coronary angiography performed in the first month, it was categorically ruled out, and intravascular ultrasound was deemed unnecessary.

In late 2018, the patient underwent extensive testing for thrombophilia and antiphospholipid antibodies [18]. Up until then, these tests were not suggested by obstetricians due to the normal first pregnancy. The genetic panel included G1691A (Factor V Leiden), 20210 G>A (Factor II, Prothrombin), 4G/5G (PAI-1 inhibitor of plasminogen activator), I/D (ACE), Val34Leu (Factor XIII), c.677C>T and c.1298A>C (MTHFR, methylenetetrahydrofolate reductase), and 66A>G (MTRR, 5-methyltetrahydrofolate-homocysteine methyltransferase reductase). The following genetic mutations were identified: homozygosity for 4G/5G PAI-1, homozygosity for I/D ACE, heterozygosity for C/T c.677C>T MTHFR, and heterozygosity for A/T c.1298A>C MTHFR. All of the tested antiphospholipid antibodies (anticardiolipin IgG, anticardiolipin IgM, antiprothrombin antibodies IgG, antiprothrombin antibodies IgM, anti-beta 2 GP1 IgM, anti-beta 2 GP1 IgG) were within normal range. She was consulted by a hemostasiologist, and the mutations she had were not considered high risk for hypercoagulability. She was advised, however, to use anticoagulant prophylaxis with low-molecular-weight heparin in case of any subsequent pregnancies. In light of the diagnosed thrombophilic mutations and the patient’s low bleeding risk, we also recommended extended treatment with low-dose aspirin [19,20].

### 2.5. A Year Later

**Figure 3:**
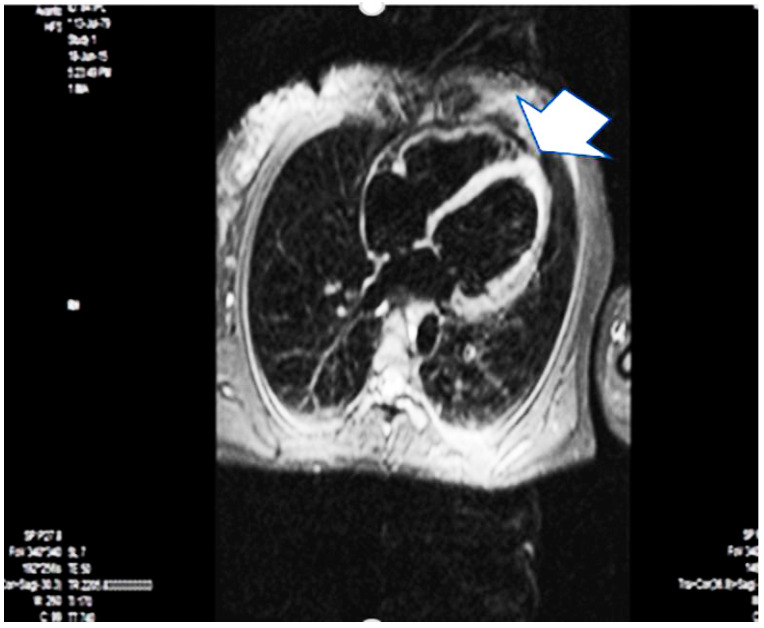
Magnetic resonance imaging–one year later—the arrow points apical fibrosis (scarring). Arrow is apical fibrosis.

A year later, the patient underwent magnetic resonance imaging due to her wishes for another pregnancy. It confirmed the dilation (EDV 180 mL, ESV 89 mL) and borderline LVEF (50%) but revealed subendocardial and transmural late gadolinium enhancement in the hypo-akinetic apical zone, consistent with postischemic fibrosis with non-vital myocardium in the region of the left anterior descending artery. This typically indicates an old myocardial infarction in this region, and since the event had a strong time relationship with the pregnancy, her diagnosis was changed to PAMI, considering the information from other reports and cases [21,22,23,24,25] (Figure 3).

The possibility of myocardial infarction with non-obstructive coronary arteries (MINOCA) was initially considered. The presence of diffuse left ventricular remodeling suggested PPCM. CMR is the gold standard for determining volumes, ejection fraction, and myocardial tissue structure. The demonstration in the patient of a sustained apical myocardial infarction (the stroke volume is derived primarily from the apex of the left ventricle), borderline ejection fraction, and complex ventricular extrasystoles classified the patient as high-risk for the planned cesarean section. Before delivery, the anesthesiologist and obstetrician-gynecologist mapped out possible scenarios for the planned cesarean section in the setting of PAMI. The patient gave informed consent. This again confirmed the need for low-dose aspirin for secondary prevention, which the patient was already taking in accordance with typical guidelines [26,27,28,29,30,31].

### 2.6. Patient Follow-Up

**Figure 4:**
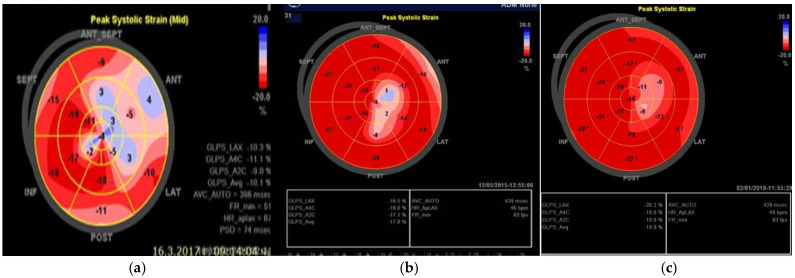
Global longitudinal strain. (**a**) in the beginning (GLS mean—10.1%). (**b**) in the third year (GLS mean—17.8%). (**c**) in the fifth year (GLS mean—19.9%) after the initial incident.

For the last five years, the patient was monitored every 6 months with EKG, echocardiography, and 24 h Holter EKG. The EKG showed no changes. Echocardiography demonstrated a complete normalization of the LVEF around 50%. Segmental abnormalities of the apex remained, with compensatory hyperkinetic contraction of the basal segments of the left ventricle sustaining a normal ejection function. Mean global longitudinal strain over time reached the normal ranges. Figure 4 contains the results from the global longitudinal strain mean from the third and fifth year after the initial event. The last mean global longitudinal strain nearly reached normal values—19.9%.

24 h EKG monitoring of the cardiac rhythm showed persisting ventricular ectopic activity up to Class IVa by Lown. This required adequate dosing of the beta-blocker. Later, ranolazine 750 mg was added twice daily; of interest was the antiarrhythmic effect of the latter in addition to the beta-blocker. Diuretics were removed from the therapeutic algorithm during the second year. The patient has excellent functional capacity—works actively and takes care of her children.

Table 1 presents the patient’s key events sequentially over time.

## 3. Discussion

PPCM is generally considered a diagnosis of exclusion, i.e., all other reasons that could lead to a similar phenotype of cardiac dysfunction, including PAMI, must be ruled out beforehand. Not much is certain about the mechanisms leading to PPCM, but it is a well-known fact that it is strongly associated with pregnancy and that it can occur up to five months post-delivery [16,17]. Our patient had some of the typical risk factors for PPCM—age, abortion, infection, multiparity—and therefore this seemed to be the case initially. Residual segmental pathology, however, is not described in the literature as a typical consequence. Similarly, pain, which was the initial presentation and also reoccurred in the active phase of the disease, does not seem to be a common symptom of PPCM. The complications that she suffered—significant reduction in left ventricular systolic function, malignant ventricular arrhythmias, even the pathological appearance of her ECG after the event—cannot with certainty be considered pathognomonic for either of the two pathologies. The identified thrombophilic mutations raise suspicion for thrombosis as the underlying mechanism, possibly not discovered due to the fact that the angiography was performed a month later. In this particular case, our conviction that this is the more rare pathology—PAMI, initially imitating PPCM—is reinforced by the findings of the cardiac magnetic resonance (CMR), which have the typical appearance of a transmural myocardial infarction with ischemia present subendocardially. For the sake of thoroughness, we could consider a coexistence between the two conditions, based on the fact that they share common risk factors and do not have a completely studied pathogenesis. PPCM is usually characterized by overt heart failure. The mechanism for developing acute myocardial infarction in a large number of these cases is different from other cases of ischemic heart disease. Coronary artery spasm associated with a probable hypercoagulability state (identified thrombophilic mutations) and undergoing spontaneous fibrinolysis was the most likely mechanism in this case, as well as in other cases, and there is a possibility that it may be based on high levels of progesterone [32,33,34,35]. The supposed mechanisms of the myocardial infarction that occurred remain hypothetical. The presented laboratory results—very high leukocytes, low magnesium, and high troponin—are from the initial examination, in the context of severe intoxication with a dead fetus. In the differential diagnostic plan, infection and dyselectrolytemia were excluded.

The initially highly elevated troponin levels were interpreted in relation to the complex state associated with the stillborn fetus. The very rapid normalization of troponin did not support myocardial infarction at onset. The identified thrombophilic mutations (homozygous PAI-1 4G/5G, etc.) were not considered “high risk” by the hemostasiologist, but anticoagulant prophylaxis was recommended. The administration of antithrombotic prophylaxis in this case was based on the patient’s history itself being a major risk factor.

The presented case has some limiting factors complicating interpretation. First, coronary angiography was performed one month after symptom onset. In making this decision, subjective factors related to stabilization of left-sided heart failure on the part of the patient played a role. This delay hampers assessment of prior coronary thrombosis or spasm. Second, CMR was performed after one year because the patient missed follow-up examinations for a long time; advice to abstain from a new pregnancy was the likely reason.

A detailed presentation of similarities and differences between PPCM and PAMI was made. The two nosological entities share common risk factors—age, consecutive pregnancies, preeclampsia. They have fewer similarities in mechanisms, with inflammation being one. There are also similarities in complications: ECG changes consistent with ischemia, malignant arrhythmias, and sudden cardiac death. In both conditions, the risk of recurrence is higher in subsequent pregnancies. According to literature, PPCM is associated with fetal death, abortion, and fetal microchimerism as mechanisms. The presented case, in contrast, demonstrates that this is not always the case. PAMI in a stillborn fetus should not be underestimated as a diagnosis. Another important conclusion from this case is the need to perform multimodal diagnostics (echocardiography, coronary angiography, magnetic resonance imaging) promptly, as they provide complementary information crucial for adequate therapy.

The initial diagnosis was PPCM, due to several reasons: stillbirth, second pregnancy, diffuse hypokinesia of the left ventricle, and normal coronary angiography. The subsequent finding of residual apical dyskinesia contradicted the PPCM diagnosis and prompted magnetic resonance imaging. The latter changed the diagnosis due to visualization of apical fibrosis—scarring. The decision about a new pregnancy was discussed many times. On one hand, there were medical risks associated with recurrence of PAMI and persistent complex ventricular arrhythmias. On the other hand, the obstetrician-gynecologist’s opinion was that the uterus was completely restored, along with the patient’s strong desire. In this case, the latter prevailed in the decision to conceive again, which was not a medical recommendation. This case shows that the decision to become pregnant again after PAMI should be individualized.

A history of previous acute myocardial infarction is not a formal contraindication for pregnancy, and the decision of getting pregnant again should be individualized [36,37]. In the current case, the decision to undergo a new pregnancy was made by the patient (a strongly desired pregnancy) without the consent of the monitoring cardiologist. The main reason was that the infarction area occupying the apex partakes most in the ejection volume of the heart. Also, PAMI has a risk of recurrence. And lastly, high-grade ventricular extrasystoles are not easily controlled with treatment. Data from literature are very contradictory. Half of the cases describe a fatal end in a second pregnancy, while others report no complications [27,38]. There are 18 reported women in this setting who had pregnancies with no complications [26,38]. All of them were evaluated previously by echocardiography, coronary angiography, and Holter monitoring to evaluate ejection fraction, coronary pattern, and presence of ischemia. The average time elapsed from the acute myocardial infarction to pregnancy is from one to two years [26,38]. The presented case had a second pregnancy during the third year after the myocardial infarction. Despite the relatively favorable outcome so far, we are still concerned about the long-term effect on the patient’s left ventricular function and risk of sudden cardiac death due to the significant arrhythmic burden, as mentioned in the literature [39,40,41,42].

## 4. Conclusions

A key message for clinicians from this case is that multimodal imaging plays a fundamental role in differentiating PPCM from PAMI. Misdiagnosis can have serious consequences for prognosis and counseling regarding the risks of a new pregnancy. It is important that multimodal imaging is performed promptly to enable adequate therapy and recommendations. The case also highlights the importance of individualized multidisciplinary management and close follow-up to optimize outcomes in complex cases where diagnoses such as PPCM and PAMI may overlap or evolve.

## Figures and Tables

**Table 1:** Timeliness summarizing key events.

in the beginning	1. second pregnancy last month 2. cardiac arrest 3. emergency cesarean section due to a stillbirth
first day	1. no cardiac sighs 2. normal echocardiography—EF = 60%
third day	1. acute left side heart failure symptoms—cardiac asthma 2. marked reduction EF = 30% 3. treatment as in heart failure with reduced EF—torasemide 5 mg, carvedilol 2 × 3.125 mg, ramipril 2.5 mg, aspirin 100 mg
first month	1. no cardiac sighs 2. EchoC-normal EF, but persist regional apical wall motion abnormalitica 3. coronary angiography—normal 4. Initially diagnosis—perypartal cardiomyopathy 5. treatment with torasemide 5 mg, carvedilol 2 × 3.125 mg, and ramipril 2.5 mg
first year	1. no cardiac sighs 2. nuclear magnetic resonance imaging of the heart- EF- 50% 3. data on the previous apical myocardial infarction 4. Finally diagnosis peripartum myocardial infarction 5. Therapy with beta blocker, ACE inhibitor, aspirin
1 year and 2 months	1. she became pregnant 2. without clinical signs 3. all medications are stopped except the beta blocker and aspirin
1 year and 11 months	1. cesarean section 2. without clinical and echocardiographic evidence of heart failure 3. complex ventricular arrhythmias 4. beta blocker, ranolazine, alternation of aspirin or low molecular weight heparin
monitoring 2, 3, 4, 5 years	1. no clinical signs of heart failure 2. normal ejection fraction 3. persistent complex ventricular arrhythmias 4. therapy—beta blocker, ranolazine and aspirin

## Data Availability

All data are in Clinic of Cardiology, Medical University, Plovdiv, Bulgaria.

## Abbreviations

The following abbreviations are used in this manuscript:

PPCM	Peripartum Cardiomyopathy
PAMI	Pregnancy-Associated Myocardial Infarction
LVEF	Left Ventricular Ejection Fraction
ECG	Electrocardiography (Electrocardiogram)
RR	Blood Pressure (Riva-Rocci, mmHg)
LV	Left Ventricle
RV	Right Ventricle
TDD	Telediastolic Diameter
TSD	Telesystolic Diameter
EDV	End-Diastolic Volume
ESV	End-Systolic Volume
EF	Ejection Fraction
BNP	B-type Natriuretic Peptide
CMR	Cardiac Magnetic Resonance (Imaging)
MRI	Magnetic Resonance Imaging
PAI-1	Plasminogen Activator Inhibitor-1
ACE	Angiotensin-Converting Enzyme
MTHFR	Methylenetetrahydrofolate Reductase
MTRR	5-methyltetrahydrofolate-homocysteine Methyl Transferase Reductase
GP1	Glycoprotein 1 (anti-beta 2 GP1 antibody)
SCAD	Spontaneous Coronary Artery Dissection
ECG-Holter	24-Hour Electrocardiography Monitoring
C-section	Cesarean Section
DOAC	Direct Oral Anticoagulant
IV	Intravenous or Intraventricular
WHO	World Health Organization
